# Human Somatosensory Processing and Artificial Somatosensation

**DOI:** 10.34133/2021/9843259

**Published:** 2021-07-02

**Authors:** Luyao Wang, Lihua Ma, Jiajia Yang, Jinglong Wu

**Affiliations:** ^1^Beijing Advanced Innovation Center for Intelligent Robots and Systems, Beijing Institute of Technology, Beijing, China; ^2^School of Mechatronical Engineering, Beijing Institute of Technology, Beijing, China; ^3^Graduate School of Interdisciplinary Science and Engineering in Health Systems, Okayama University, Okayama, Japan

## Abstract

In the past few years, we have gained a better understanding of the information processing mechanism in the human brain, which has led to advances in artificial intelligence and humanoid robots. However, among the various sensory systems, studying the somatosensory system presents the greatest challenge. Here, we provide a comprehensive review of the human somatosensory system and its corresponding applications in artificial systems. Due to the uniqueness of the human hand in integrating receptor and actuator functions, we focused on the role of the somatosensory system in object recognition and action guidance. First, the low-threshold mechanoreceptors in the human skin and somatotopic organization principles along the ascending pathway, which are fundamental to artificial skin, were summarized. Second, we discuss high-level brain areas, which interacted with each other in the haptic object recognition. Based on this close-loop route, we used prosthetic upper limbs as an example to highlight the importance of somatosensory information. Finally, we present prospective research directions for human haptic perception, which could guide the development of artificial somatosensory systems.

## 1. Introduction

Benefiting from developments in the field of cognitive neuroscience, we have been able to learn more about how the human brain perceives external information, including recognizing (being aware of), organizing (gathering and storing), and interpreting (binding to knowledge) objects. Perception is a remarkable human ability, normally involving five senses: vision, audition, touch, smell, and taste. Over the past two decades, artificial sensory systems have gathered much attention and have obtained significant achievements in imitating human visual and auditory senses, such as computer vision and speech recognition [1]. The corresponding products have played a great role in industrial production and daily life. To date, vision and audition systems have been well studied, and touch has been addressed only more recently. Understanding touch will benefit the artificial system to interact directly with objects and obtain information (e.g., texture, temperature, and softness).

**Figure 1:**
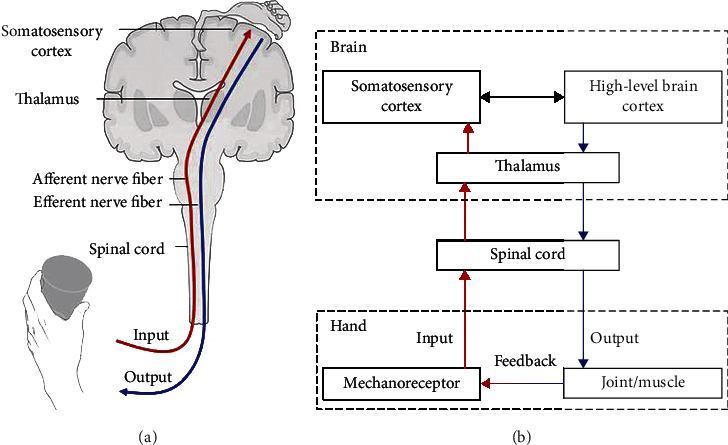
Human somatosensory pathway. (a) Schematic diagram. (b) Flow chart. The red line is the ascending somatosensory pathway, which refers to the neural pathways by which haptic information from the peripheral mechanoreceptor is transmitted to the cerebral cortex. The blue line is the descending motor pathway, which refers to the pathways by which motor signals are sent from the brain to lower motor neurons in joint and muscle.

Touch could protect our bodies because many receptors are on the skin, which covers the whole body and detect harmful stimuli [2]. Touch is the first of the fetal senses to come into play in the womb, which is an effective way of experiencing social behaviour and communicating emotions (e.g., holding hands and hugging) [3, 4]. In addition, haptic perception plays an important role in object recognition and manipulation. Hands have both receptor and executive functions, and they have the highest spatial discrimination and ability to manipulate objects in fine detail [5]. Hands not only transmit external information to the brain through afferent nerve fibers (ascending somatosensory pathway) but also receive real-time adjustment from the brain through efferent nerve fibers (descending motor pathway) [6, 7] (Figure 1). Bionic hands integrated with artificial skin could perceive different dimensions of external haptic information, which have great potential in communicating with complex environments, recognizing objects, and even engaging in social interaction [8]. In addition, artificial somatosensory systems simulating human somatosensory pathways have more extraordinary applications, such as manual palpation and prosthetic upper limbs, potentially bestowing lost sensory feelings to amputees by “interfacing” with the brain and the body [9]. Artificial somatosensory systems require a comprehensive understanding of the mechanisms of the human somatosensory system. The intrinsic patterns of the interplay between the human somatosensory system and activity-dependent factors are central to the development of artificial somatosensory systems [10].

The human somatosensory system serves three major functions: exteroceptive (perceiving stimuli outside of our body), interoceptive (perceiving stimuli inside of our body), and proprioceptive (controlling body position and balance) functions. On the ascending somatosensory pathway, the perception of basic physical quantities in the external environment is mainly based on the exteroceptive system. Haptic information needs to be transmitted from peripheral mechanoreceptors through the spinal cord, dorsal column nuclei, and ventral posterolateral nucleus (VPL) of the thalamus to the primary somatosensory cortex (S1) and higher association areas, such as prefrontal cortex [11]. We encounter no difficulty in differentiating different objects by touching them. For example, we could recognize two cups of different materials, shapes, and sizes but use the word “cup” to name these two objects. This process involves the extraction of different stimulus features in the human skin. Then, primary brain areas and higher association areas combine these features and integrate them with prior knowledge [12–19]. On the descending motor pathway, the interaction of exteroceptive and proprioceptive is important. Following the principle developed from visual system, previous studies reported that the processing pathway for object recognition was separated for action planning guided by somatosensory information [20]. However, there is now clear evidence for similar underlying neural networks related to these two pathways, especially for the processing of haptic object recognition [21, 22]. Disruptions along the somatosensory pathway result in poor muscle control and object manipulation [23].

Many major strides have been made in the past 50 years in exploring how the brain encodes somatosensory information and which relevant pattern activation could be reproduced to elicit similar sensations [24]. However, there is still much neural coding that we do not understand, which prevents us from advancing any further in possible applications. Researchers summarized models of five higher-order brain networks related to haptic information processing, including haptic object recognition and memory, body perception, body ownership, affective processing, and action [20]. Based on previous studies of the human perception, the contribution of our review is that we provide a comprehensive framework of the somatosensory processing pathway from peripheral stimuli on the skin to the brain cortex, including characteristics of low-threshold mechanoreceptors, organization principle and brain areas related to haptic perception. Each part provides theoretical basis for the applications of artificial somatosensation, such as tactile sensor design, artificial skin, and bionic hands (prosthetic upper limbs). Finally, we provide future directions for the human somatosensory system, which is fundamental to artificial somatosensation. The development of artificial somatosensation benefits applications in wearable electronic devices and devices used in the biomedical field, human-computer interaction, intelligent robotics, and other fields. Thus, basic science will inform the development of next-generation artificial somatosensory systems, and artificial somatosensory systems will in turn lead to new insight into basic science.

## 2. Physical Quantity Recognition

There are four kinds of mechanoreceptors in the human glabrous skin, and their responses to the haptic stimulus are the input of the somatosensory system. Various basic physical quantities constitute the elements of haptic stimulus, such as location, frequency, and pressure. The combination of different physical quantities further forms the characteristics of the object. The process of object recognition is the integration of basic physical quantity and maybe affected by other factors (e.g.m experience and emotion). We generalized mechanoreceptors and their corresponding afferent fibers in the human skin and then summarized haptic sensors in the artificial skin. Both were used to detect the basic physical quantities of touch and transmit haptic input information. The response properties of neurons and fibers in the human skin provide a theoretical basis for haptic sensors.

### 2.1. Low-Threshold Mechanoreceptors on the Human Skin

**Figure 2:**
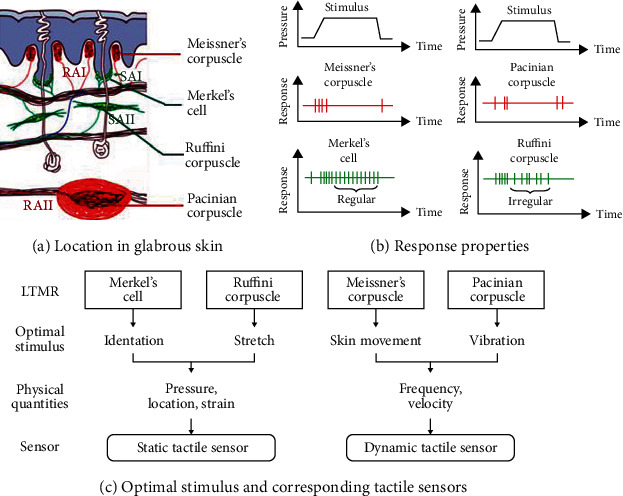
A comparison of four types of low-threshold mechanoreceptors (LTMRs). (a) Location of the LTMRs in the glabrous skin. (b) Stimulus response properties. (c) Optimal stimulus for each LTMR and corresponding haptic sensor.

Within the exteroceptive somatosensory system, the perception of innocuous and noxious haptic sensation relies on low-threshold mechanoreceptors (LTMRs) and high-threshold mechanoreceptors (HTMRs). The LTMRs react to innocuous mechanical stimulation while the HTMRs respond to harmful mechanical stimuli, such as pain. Pain and touch are intricately related; thus, a large portion of our somatosensory system is devoted to deciphering which is harmful [25, 26]. There are four types of LTMRs in the glabrous skin: Merkel cells, Ruffini ending, Meissner corpuscle, and Pacinian corpuscle (Figure 2(a)). They transfer complex haptic information (deformation of tissues—skin, muscles, tendons, ligaments, or joints) into neural codes [6, 24]. The anatomical and physiological characteristics of these LTMRs are different and are integrated into the state of the contacted objects (Figure 2(b)). In addition, they are innervated by four different classes of afferent fibers [27, 28]. According to the size of their receptive fields (RFs), they could be classified into type I (close to the surface of the skin with small and clearly defined RFs) and type II (deeper in the skin and have large RFs with ill-defined boundaries). Furthermore, each type could be further classified based on their speed of adaption, ranging from slowly adapting to rapidly adapting. Their firing patterns in responding to skin indentations are quite different: slowly adapting produce a sustained firing while rapidly adapting response only at the onset and offset of the indentation. In addition, A*δ* and c-haptic fibers were found in the hairy skin, which respond most strongly to temperature and affective touch. In this paper, we mainly focus on the glabrous skin which plays a key role in object recognition.

Slowly adapting type I (SAI) afferent fibers supply clusters of Merkel cells. Merkel cells respond maximally to corners, edges, and curvatures of objects and have high spatial resolution, which endows them with the ability to transmit stimulus position and reconstruct acute spatial images of haptic stimuli [29]. The Ruffini endings yield a sustained response to skin indentation with different interspike intervals, which are associated with slowly adapting type II (SAII) fibers. They are two to four times more sensitive to skin stretch and changes in hand and finger shape than Merkel cells [30]. Rapidly adapting type I (RAI) fibers innervate Meissner corpuscles. One of the functions of Meissner corpuscles is to detect and determine the scale of low-frequency vibrations. They may also play an important role in movement detection across the skin and grip control and would be involved in situations such as keeping the object you are holding from slipping [31]. Conversely, Pacinian corpuscles innervated by rapidly adapting type II (RAII) fibers that are constantly firing to detect high-frequency vibration convey information about the texture of an object held in the hand [32]. These fibers are the basis of touch perception and serve as a reference for the artificial skin with haptic sensors to obtain information about objects.

### 2.2. Haptic Sensors on the Artificial Skin

For the artificial somatosensory system, haptic sensors act as the mechanoreceptors. They could mimic the response properties of LTMRs in the human skin to transfer information about grasped objects, such as pressure, frequency, hardness, shape, slip, and texture. They have been developed and applied in routine life or industrial scenes since the early 1970s [33]. According to the transduction mechanisms, haptic sensors can be classified into capacitive [34], piezoresistive [35], piezoelectric [36], optical [37], and magnetic sensors [38]. To recognize the properties of objects, we can interact with them through static or dynamic touch (Figure 2(c)). In static touch, there are different haptic sensors for measuring pressure based on the response properties of LTMRs innervated by SAI and SAII [39–41]. By extracting shape features from pressure distributions, haptic sensors can recognize the shape of contact objects [42]. Lee et al. explored a stretchable crossreactive sensor matrix that could discriminate multimodal haptic sensation, including strain, pressure, flexion, and temperature [43].

In dynamic touch, texture and roughness can be detected by the skin sliding across the surface of objects, which are critical properties for recognizing objects. These kinds of characteristics are related to high-frequency vibration, which induces responses from RAIs and RAIIs. Choi et al. explored the artificial skin imitating human epidermal fingerprint ridges and the epidermis to distinguish various textures [44]. Gong et al. constructed a pneumatic haptic sensor to detect force, vibration, and slippage based on changes in the pressure of the air bladder, which could perceive objects' softness and roughness [45]. Gastaldo et al. focused on a tensor-based approach to classify three touch modalities, including brushing, sliding, and rolling [46]. Researchers adopted a two-layer model of spike-based neuromorphic encoding of haptic stimuli to create a haptic feature extractor [47]. This model can decode geometric edge orientations under different sensing forces and velocities. In addition, some researchers have focused on improving different machine learning algorithms, such as *K*-means clustering [48] and backpropagation artificial neural networks [49], to classify object surfaces according to contact forces and slippage in haptic sensors. Researchers from different fields have made great efforts to explore new techniques and materials for advanced haptic sensors. Zou et al. summarized fabrication technologies that have been developed and that contribute to these hardware applications [50].

Apart from detecting physical information about objects, learning is an important subcomponent for recognizing objects. It involves storing haptic information in short-term memory (STM) and long-term memory (LTM) and associating it with object knowledge. STM is lost quickly without repeated stimulation, while LTM is related to permanent memory. The plasticity or synaptic modulation of biological neural systems results in various forms of memory. Inspired by the somatosensory system, neuromorphic circuits emerged, which have been used on the artificial skin [51]. The state of internal resistance of two-terminal memristive devices could represent the history of voltage, which has gained attention [52, 53]. Some researchers have reported the long-term storage of pressure patterns related to haptic sensors with nonvolatile memory, which is similar to LTM. Tan et al. reported an optoelectronic spiking afferent nerve that could not only detect pressure but also recognize and memorize handwritten alphabets and words [54]. Furthermore, combining STM and LTM enables haptic devices to have a multilevel forgetting process and to memorize a rich amount of information. Wu et al. developed haptic sensors with the capacity to mimic learning and memory based on the principle of a triboelectric nanogenerator. The authors classified the film into STM and LTM types, which could produce signals according to current and history pressure stimulations [55]. Kim et al. presented an intelligent haptic perception device that could process short- and long-term plasticity in parallel [56].

## 3. Somatotopic Organization Principle

**Figure 3:**
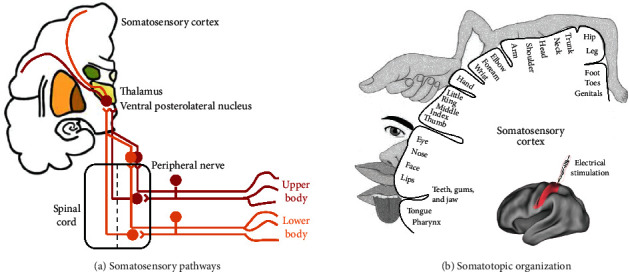
Somatosensory-related areas. (a) General organization of the somatosensory pathway. (b) Somatotopic map of the primary somatosensory cortex.

Haptic information received by mechanoreceptors ascends through the spinal cord and ventral posterolateral nucleus of the thalamus to the S1 (Figure 3(a)). Along this pathway, somatotopy (topographic organization) is an important guiding principle for the sensory fiber organization along the dorsal root ganglion, the medulla, and the VPL nucleus of the thalamus and is finally projected in the S1. Kohonen used this topography principle to develop a well-known self-organizing feature map (SOM) algorithm [57]. Artificial skin based on a SOM can self-calibrate by automatically learning the structure and spatial distribution of its sensors [58].

### 3.1. Somatotopic Map in the Human Brain

To recognize an object, it is critical to know which parts or fingers of our hand are in contact with the object. When a finger contacts an object, a specific population neuron in the cerebral cortex is activated. Behavioural studies found that body parts are segmented with joints as boundaries [59]. A somatotopic map establishes a mapping between external haptic information input and brain activation. In other words, through the somatotopic map, we could know which finger is in contact with an object. This somatotopic representation that occurs in the S1 was first described in the 1930s by Penfield [60]. Intraoperative electrical cortical stimulation was applied to epileptic patients, and this stimulation could induce sensations at specific locations in the patient's body. The projected location varied with the location of the stimulating electrode, which was visualized in the form of “homunculi” (Figure 3(b)). This systematic organization could also be detected by fMRI [61, 62]. Numerous studies have confirmed that hands occupy the largest areas, probably because of the need to perform refined functions in daily life [63, 64].

Furthermore, the S1 could be subdivided into area 3a, 3b, 1, and 2. Studies found multiple somatotopic maps in the S1, which showed mirrored patterns at the boundaries of these areas (i.e., proximal-to-distal phalanx representation is posterior to anterior in area 3b but anterior to posterior in area 1) [65]. The somatotopic maps were slightly different across these 4 subregions. Studies have revealed that the amount of overlap between finger representations in the area 1 was larger than that in area 3b and even responded to up to five fingers, and neurons in areas 3b and 1 responded to light touch. Neurons in area 2 are more complex than those in areas 3b and 1, as neurons in area 2 respond to both touch and proprioception. In addition, neurons in area 3a respond primarily to movements of the joints [66]. Apart from the location information, the S1 early processes simple features and detects the direction or velocity of a moving target over the surface [67]. Then, multidimensional features are combined in higher level areas to provide information about the objects or integrate them in a representation of our body.

Based on the somatotopic organization principle and neuronal response characteristics, researchers used intracortical microstimulation (ICMS) to restore touch by delivering trains of electrical pulses directly to the somatosensory areas of the brain. It has been successfully used in animals, and it could guide animals to discriminate the location pokes, feel different levels of pressure, determine contact timing, and even detect higher-level features by stimulating different neuronal populations [68]. In addition, Flesher et al. first implanted the ICMS in a patient with a long-term spinal cord injury in the hand area of the S1 [69]. The interface conveyed information about grasped objects by creating a systematic mapping between haptic information and neuronal activation in the brain, which could be used to guide user behaviur. The results showed that haptic sensations with naturalistic characteristics (e.g., pressure) could be perceived and evoked stably after a few months. This could be used as the basis for the implementation of artificial somatosensory systems in prosthetic upper limbs for patients with spinal cord injury. Researchers could modulate different types of stimuli, which could be used to convey more haptic features associated with grasped objects in the future. One of the challenges is cortical plasticity, which could influence somatotopic representation. Using 7 Tesla fMRI technology, rapid reorganization in the somatosensory cortex was revealed after 24 hr gluing manipulation [70]. Although the change is not as dramatic as it might seem, whether the functional properties of the neurons in the cortex change after deafferentation should be further investigated [68]. This is important determining for how to apply feature-specific stimulation to recover somatosensation.

### 3.2. Self-Organizing Feature Map on the Artificial Skin

Based on somatotopic information, Pugach et al. trained the artificial skin to distinguish different surface shapes, such as squares, circles, and prisms [71].This artificial skin was also useful for reconstructing 3D haptic surfaces [72]. Approximately 17,000 cutaneous afferents innervate the human hand, with densities peaking at approximately 240 units/cm^2^ at the fingertips [73]. Due to the limitations of sensor size and function, the density is even larger in the artificial skin [74]. The integration of a large amount of haptic sensory information is a significant challenge in artificial skin devices, which require complicated multiplayer architectures. To solve this problem, Bergner et al. combined modularity and SOM in an artificial skin system and proposed an event-driven approach to manage the large amount of information [75]. This significantly contributes to the feasibility of large-area haptic applications. We perceive that two stimuli are farther apart when across the wrist than when they were both on palm [76]. This representation influences the perception of spatial haptic stimuli and is helpful for coordinating the interaction across fingers when they are working together. Liu et al. proposed a recognition method based on a joint kernel to solve the problem of interference across multiple fingers when they contact objects at the same time [77].

## 4. Somatosensory Processing for Action

After extracting and organizing different stimulus features in the human skin and S1, somatosensory areas interact with other brain areas to achieve high-level haptic perception, such as object recognition and action guidance. Touch and movement are closely related and interact with each other. In the process of the haptic object recognition, the two form a closed-loop route. Somatosensory provides information about the location of the body and limbs, which guides the plan of action and posture adjustment. Here, we summarized areas for object recognition and action-related processing. In addition, we used the prosthetic upper limb as an example and demonstrated the benefits of somatosensory feedback in practical applications based on the present understanding of the human brain.

### 4.1. Areas Related to Haptic Perception in the Human Brain

**Figure 4:**
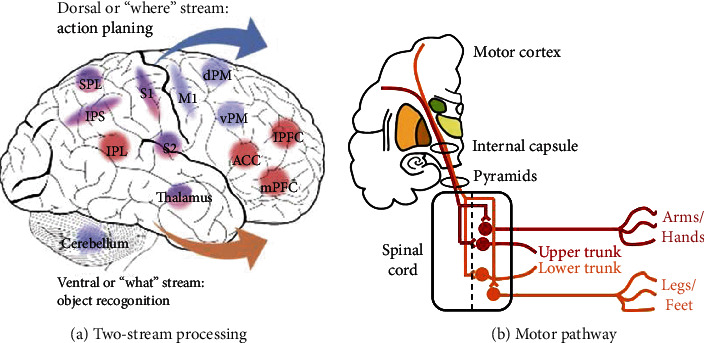
Somatosensory processing for action. (a) Two-stream processing in the somatosensory system. (b) General organization of the motor corticospinal pathway. SPL: superior parietal lobe; IPS: intraparietal sulcus; IPL: inferior parietal lobe; S1: primary somatosensory cortex; S2: secondary somatosensory cortex; M1: primary motor cortex; dPM: dorsal premotor cortex; vPM: ventral premotor cortex; ACC: anterior cingulate cortex; lPFC: lateral prefrontal cortex; mPFC: medial prefrontal cortex.

There are reciprocal connections between the S1 and the secondary somatosensory cortex (S2). Neurons in the S2 have larger receptive fields which span multiple fingers and can even encompass both hands [78]. In addition, they could respond to various types or modalities of stimuli [79]. Previous studies confirmed that one processing stream projected from S1 via S2 to the posterior insula and frontal cortex, which is associated with object recognition and memory. In addition, there were separated stream projects to the premotor cortex and the limbic cortex via the posterior parietal cortex (PPC), which is associated with action-related processing [20] (Figure 4(a)). Similar to the “ventral and dorsal streams” in the visual and auditory systems, researchers hypothesize that the somatosensory system could also be divided into two subsystems (e.g., systems establishing information to determine “where” and” what”) [80]. However, it is obvious that these two streams interact symbiotically, especially for the processing of haptic perception, which refers to the active exploration of surfaces and objects by a moving subject, as opposed to passive contact by a static subject during haptic perception. For studies on the artificial skin, Fonseca et al. adapted the “what and Where” systems to haptic sensors [81]. The “what” system was used to recognize surface features of objects through haptic sensors, whereas the “where” system provided a description of the contact location on the skin. This improves inhand manipulation, object characteristic extraction, and feedback control.

We use fingertips to detect finer details and palms for larger surfaces of objects and specific movement postures to extract information. There are six different exploratory procedures that we perform when perceiving haptic stimuli, such as “contour following” and “enclosure,” which are used for the shape recognition [80]. Then, some information integrated together at the receptors level and some projected to the limbs and trunk area through the spinal cord (Figure 4(b)). Our brain further combines cutaneous (exteroceptive) and kinesthetic (proprioceptive) inputs for object recognition and action guidance, during which the left PPC and motor cortex are activated [82]. Penfield et al. also reported a “homunculus” in the motor cortex [60]. Recent studies found that the organization principle in the primary motor cortex was slightly different from that in the somatosensory cortex. It was dependent on whether the digits' muscles were used for different motor actions, such as grasping or retraction movements [83]. Somatosensory and motion information are closely related. Somatosensory-guided action has been reported to influence grasping movement if haptic feedback is withdrawn [84]. In individuals with intact arms and motor pathways but without somatosensory feedback, movements are slow, clumsy, and effortful. Most artificial skin studies have focused on combining haptic and proprioception information to obtain a more comprehensive understanding of the objects. Luo et al. presented a method that could link the local haptic features with kinesthetic cues to recognize object shapes [85]. In addition, Pastor et al. provided a 3D convolutional neural network to classify grasped objects through active interaction based on haptic tensors [86].

### 4.2. Somatosensory Feedback for Prosthetic Upper Limbs

Based on the understanding of the somatosensory processing pathway, prosthetic upper limbs with somatosensory information could help amputees restore the haptic function and interact with objects flexibly. Conventional prosthetic limbs can collect signals from the residual muscles in upper-limb amputees. However, the lack of somatosensory feedback makes offers poor control over these limbs and makes it difficult for individuals using them to interact with objects flexibly. Humans could grasp objects robustly without prior knowledge of them with appropriate pressure and posture so that the object would not slip from our hand or crumble. For prosthetic limbs, somatosensory feedback must be restored to obtain information about objects intuitively and further integrated into the motor plan for object manipulation [87]. This process benefits from real-time somatosensory feedback and adjustment. Establishing electrical connection with the peripheral nervous system of amputees provides rudimentary but reliable somatosensory feedback from prosthetic limbs in activities of daily living. Researchers have proven that prosthetic limbs that utilized somatosensory feedback, including cutaneous and kinesthetic information, had higher object recognition accuracy [22].

**Figure 5:**
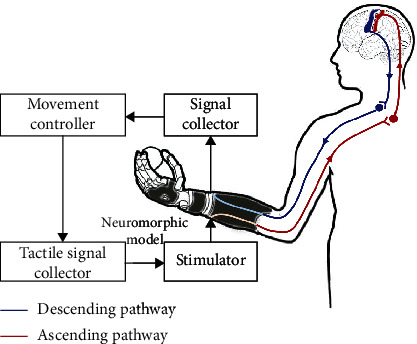
Prothesis system diagram. Haptic information from an object is transformed into a neuromorphic signal. The neuromorphic signal is used to transcutaneously stimulate the peripheral nerves of an amputee to elicit the sensory perception of touch and then ascend to the brain through the spinal cord.

Some researchers have focused on modeling aggregate afferent responses of haptic fibers to haptic stimuli, which could be used to convert signals from haptic sensors into biological patterns of electrical stimulation [88]. This kind of biomimetic encoding model could be used in peripheral nerve interfaces for prosthetic limbs. Osborn et al. developed a multilayer electronic dermis with a sense of self-preservation and the ability to automatically release an object when pain is detected; this dermis was then was applied to prosthetic limbs [89]. When grasping objects, information could be transformed into neuromorphic signals and then elicit haptic perception by transcutaneously stimulating peripheral nerves of the amputee (Figure 5). Somatosensory feedback could also be provided invasively using surgically implanted electrodes within the residual limbs. Benefitting from the neuromusculoskeletal prosthesis, movement control could be achieved by extracting signals from electrodes implanted on viable muscle tissue, and somatosensory feedback could be provided by stimulating afferent nerve fibers [87]. Apart from the peripheral nerve, epidural spinal cord stimulation is an alternative approach for somatosensory restoration of patients with proximal amputations [90]. In addition, brainstem dorsal column nuclei may be another alternative target to restore somatosensation [91]. Although researchers have been searching for the most effective stimulation targets, both invasive and noninvasive prosthetic limbs hold the potential to provide closed-loop control through ascending somatosensory processing pathways.

## 5. Concluding Remarks and Future Directions

In this review, we proposed a comprehensive framework for the human somatosensory system from the peripheral skin to the brain cortex, which provides the theoretical basis for the artificial somatosensory system (Table 1). First, we summarized the characteristics of four types of low-threshold mechanoreceptors in the glabrous skin, which could recognize basic physical quantities and are fundamental for haptic sensor design. The proprioception could provide information about the states of the muscles and limbs, which could guide motion planning. Then, haptic information is projected to the somatosensory cortex through the nerve fiber in the spinal cord; notably, the somatotopic map is the important organization principle along this pathway. This corresponds to the signal transmission in the artificial somatosensory system. In addition, high-level cerebral cortex participated in object recognition and action guidance, which provided theoretical basis for artificial intelligence. Using somatosensory feedback to form closed-loop control systems is promising and meaningful in bionic hands, such as prosthetic upper limbs.

Although there have been major advances in artificial somatosensation, there are still many obstacles in practical applications, such as the integration and processing of redundant information. In addition, improving the transduction mechanism and designing material to fit the skin tissue (e.g., soft sensors with better stretching and strengthening) could offer improved biocompatibility and interpretability. Faced with the application requirement of artificial somatosensation, understanding how touch signals are encoded and transferred through the somatosensory processing pathway is important. An fMRI study found that natural hand use shapes the relative arrangement of finger-specific activity patterns in the sensory-motor cortex [92]. Understanding haptic information in daily life is necessary for improving the design of artificial somatosensory systems. Shao et al. presented a wearable haptic sensing array covering the whole hand, which could capture human haptic signals during natural interactions [93]. In the future, researchers could try to reproduce biological activity patterns that naturally evoked during everyday interactions with objects. However, there are still many unresolved questions about the somatosensory pathway that are worthy investigating and driving the development of artificial somatosensation.

**Figure 6:**
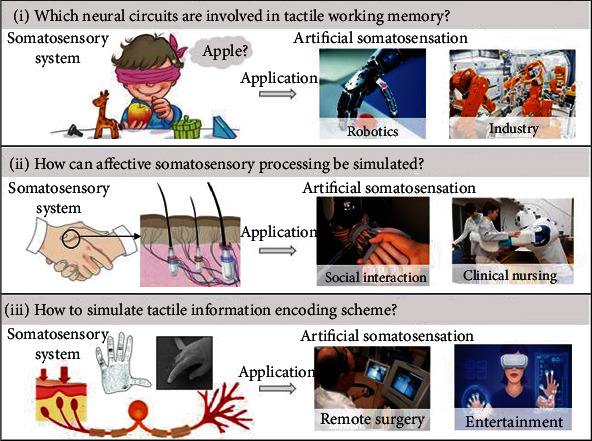
Future direction. We proposed three unresolved questions about the somatosensory pathway including the processing of haptic working memory, affective information, and somatosensory encoding scheme. The new findings of the human somatosensory system could promote the development artificial somatosensation and could be applied in more fields.

First, which neural circuits are involved in haptic working memory? An important question is the time course of activation and causation across different areas. Although we designed haptic sensors that could store haptic information, they could store only simple haptic physical quantities. Understanding the dynamic collaboration across areas could help us design a better artificial somatosensory system, which could be applied in robotics and industry. Technologies with high time resolution, such as electroencephalography and magnetoencephalography, possibly combined with transcranial magnetic stimulation may provide some insight into these questions. Second, how can affective somatosensory processing be simulated in the artificial skin? The sensory channel for the positive affective aspect of touch is the c-haptic system, which contains LTMRs connected to slow-conducting unmyelinated fibers. Affective touch activates the bilateral network through c-fibers in the hairy skin, including the posterior and anterior insula, the postcentral primary and secondary somatosensory cortex, the putamen, the thalamus, the frontal operculum, and the medial prefrontal cortex [20]. However, research conveying emotion and social interaction in artificial somatosensory systems is still in its infancy. Understanding the mechanism of affective touch could help us design products used in social interaction and clinical nursing. Third, how to simulate haptic information encoding scheme, which could be used on remote transmission? With the development of Internet and virtual reality technology, the haptic sensors could connect remote or virtual object, providing haptic feedback to the human skin. For example, in robotic minimally invasive surgery, feedback from haptic sensors is crucial to recognize diseased sites and preserve health tissues, especially in remote surgery. With the development of basic scientific theories, artificial somatosensation will increasingly mimic its natural counterpart and could be applied in more fields (Figure 6).

## Figures and Tables

**Table 1:** Basic concepts of the human and artificial somatosensory system.

Human somatosensory system		Artificial somatosensory system	
Term	Interpretation	Term	Interpretation
Mechanoreceptor	Sensory cells, including Merkel cells, Ruffini endings, Meissner corpuscles, and Pacinian corpuscles, respond to mechanical pressure or distortion	Haptic sensor	Measures information arising from physical interaction with the environment. These sensors are generally modeled after the biological sense of cutaneous touch
Proprioception	Provides information about the body's position and the state of the muscles and limbs	Motion planning	Finding a sequence of valid configurations that moves the object from the source to the destination
Nerve fiber	Afferent nerve fibers and efferent nerve fibers that conduct action potentials away from the nerve cell body	Signal transmission	The transduction mechanisms of haptic sensors could be classified into capacitive, piezoresistive, piezoelectric, optical, and magnetic
Cerebral cortex	Neural integration in the central nervous system. It plays a key role in attention, perception, memory, language, and consciousness	Artificial intelligence	Mimics “cognitive” functions that humans associate with the human mind, such as “learning” and “problem solving”

## References

[1] Martell J., Elmer T., Gopalsami N., Park Y. S. (2011). Visual measurement of suture strain for robotic surgery. *Computational and Mathematical Methods in Medicine*.

[2] Vallbo A. B., Johansson R. S. (1984). Properties of cutaneous mechanoreceptors in the human hand related to touch sensation. *Human Neurobiology*.

[3] McGlone F., Vallbo A. B., Olausson H., Loken L., Wessberg J. (2007). Discriminative touch and emotional touch. *Canadian Journal of Experimental Psychology-Revue Canadienne De Psychologie Experimentale*.

[4] Löken L. S., Wessberg J., McGlone F., Olausson H. (2009). Coding of pleasant touch by unmyelinated afferents in humans. *Nature Neuroscience*.

[5] Mancini F., Bauleo A., Cole J. (2014). Whole-body mapping of spatial acuity for pain and touch. *Annals of Neurology*.

[6] Abraira V. E., Ginty D. D. (2013). The sensory neurons of touch. *Neuron*.

[7] Lemon R. N. (2008). Descending pathways in motor control. *Annual Review of Neuroscience*.

[8] Zollo L., Di Pino G., Ciancio A. L. (2019). Restoring tactile sensations via neural interfaces for real-time force-and-slippage closed-loop control of bionic hands. *Science robotics*.

[9] Núñez C. G., Navaraj W. T., Polat E. O., Dahiya R. (2017). Energy-Autonomous, Flexible, and Transparent Tactile Skin. *Advanced Functional Materials*.

[10] Hoffmann M., Straka Z., Farkas I., Vavrecka M., Metta G. (2018). Robotic homunculus: learning of artificial Skin representation in a humanoid robot motivated by primary somatosensory cortex. *Ieee Transactions on Cognitive and Developmental Systems*.

[11] Song W. G., Semework M. (2015). Tactile representation in somatosensory thalamus (VPL) and cortex (S1) of awake primate and the plasticity induced by VPL neuroprosthetic stimulation. *Brain Research*.

[12] Gurtubay-Antolin A., Leon-Cabrera P., Rodriguez-Fornells A. (2018). Neural Evidence of Hierarchical Cognitive Control during Haptic Processing: An fMRI Study. *Eneuro*.

[13] Kassuba T., Menz M. M., Röder B., Siebner H. R. (2013). Multisensory interactions between auditory and haptic object recognition. *Cerebral Cortex*.

[14] Zhao D., Zhou Y. D., Bodner M., Ku Y. (2018). The causal role of the prefrontal cortex and somatosensory cortex in tactile working memory. *Cerebral Cortex*.

[15] Yang J., Kitada R., Kochiyama T. (2017). Brain networks involved in tactile speed classification of moving dot patterns: the effects of speed and dot periodicity. *Scientific Reports*.

[16] Yang J., Molfese P. J., Yu Y. (2021). Different activation signatures in the primary sensorimotor and higher-level regions for haptic three-dimensional curved surface exploration. *NeuroImage*.

[17] Yang J., Yu Y., Shigemasu H. (2021). Functional heterogeneity in the left lateral posterior parietal cortex during visual and haptic crossmodal dot-surface matching. *Brain and Behavior: A Cognitive Neuroscience Perspective*.

[18] Yu Y., Huber L., Yang J. (2019). Layer-specific activation of sensory input and predictive feedback in the human primary somatosensory cortex. *Science advances*.

[19] Yu Y., Yang J., Ejima Y., Fukuyama H., Wu J. (2017). Asymmetric functional connectivity of the contra- and ipsilateral secondary somatosensory cortex during tactile object recognition. *Frontiers in Human Neuroscience*.

[20] de Haan E. H. F., Dijkerman H. C. (2020). Somatosensation in the brain: a theoretical re-evaluation and a new model. *Trends in Cognitive Sciences*.

[21] Limanowski J., Friston K. (2020). Attentional modulation of vision versus proprioception during action. *Cerebral Cortex*.

[22] Schiefer M. A., Graczyk E. L., Sidik S. M., Tan D. W., Tyler D. J. (2018). Artificial tactile and proprioceptive feedback improves performance and confidence on object identification tasks. *PLoS One*.

[23] Vallar G., Ronchi R. (2009). Somatoparaphrenia: a body delusion. A review of the neuropsychological literature. *Experimental Brain Research*.

[24] Delhaye B. P., Long K. H., Bensmaia S. J. (2018). Neural basis of touch and proprioception in primate cortex. *Comprehensive Physiology*.

[25] Basbaum A. I., Bautista D. M., Scherrer G., Julius D. (2009). Cellular and molecular mechanisms of pain. *Cell*.

[26] Todd A. J. (2010). Neuronal circuitry for pain processing in the dorsal horn. *Nature Reviews Neuroscience*.

[27] Cheney P. D., Preston J. B. (1976). Classification and response characteristics of muscle spindle afferents in the primate. *Journal of Neurophysiology*.

[28] Johansson R. S., Landstrom U., Lundstrom R. (1982). Responses of mechanoreceptive afferent units in the glabrous skin of the human hand to sinusoidal skin displacements. *Brain Research*.

[29] Williams A. L., Gerling G. J., Wellnitz S. A., Bourdon S. M., Lumpkin E. A. Skin relaxation predicts neural firing rate adaptation in SAI touch receptors.

[30] Edin B. B. (1992). Quantitative analysis of static strain sensitivity in human mechanoreceptors from hairy skin. *Journal of Neurophysiology*.

[31] Torebjork H. E., Ochoa J. L. (1980). Specific sensations evoked by activity in single identified sensory units in man. *Acta Physiologica Scandinavica*.

[32] Halata Z. (1977). The ultrastructure of the sensory nerve endings in the articular capsule of the knee joint of the domestic cat (Ruffini corpuscles and Pacinian corpuscles). *Journal of Anatomy*.

[33] Chi C., Sun X., Xue N., Li T., Liu C. (2018). Recent Progress in Technologies for Tactile Sensors. *Sensors (Basel)*.

[34] Hu X., Zhang X., Liu M. (2014). A flexible capacitive tactile sensor array with micro structure for robotic application. *Science China Information Sciences*.

[35] Okatani T., Takahashi H., Noda K., Takahata T., Matsumoto K., Shimoyama I. (2016). A tactile sensor using piezoresistive beams for detection of the coefficient of static friction. *Sensors*.

[36] Maita F., Maiolo L., Minotti A. (2015). Ultraflexible tactile piezoelectric sensor based on low-temperature polycrystalline silicon thin-film transistor Technology. *IEEE Sensors Journal*.

[37] Ahmadi R., Packirisamy M., Dargahi J., Cecere R. (2012). Discretely loaded beam-type optical Fiber tactile sensor for tissue manipulation and palpation in minimally invasive robotic surgery. *IEEE Sensors Journal*.

[38] Alfadhel A., Khan M. A., Cardoso de Freitas S., Kosel J. (2016). Magnetic tactile sensor for braille reading. *IEEE Sensors Journal*.

[39] Huh T. M., Liu C., Hashizume J. (2018). Active sensing for measuring contact of thin film gecko-inspired adhesives. *Ieee Robotics and Automation Letters*.

[40] Liu C., Zhuang Y., Nasrollahi A., Lu L., Haider M. F., Chang F. K. (2020). Static tactile sensing for a robotic ectronic skin via an electromechanical impedance-based approach. *Sensors (Basel)*.

[41] Pu X., Liu M., Chen X. (2017). Ultrastretchable, transparent triboelectric nanogenerator as electronic skin for biomechanical energy harvesting and tactile sensing. *Science Advances*.

[42] Luo S., Bimbo J., Dahiya R., Liu H. (2017). Robotic tactile perception of object properties: a review. *Mechatronics*.

[43] Lee J. H., Heo J. S., Kim Y. J. (2020). A behavior-learned cross-reactive sensor matrix for intelligent skin perception. *Advanced Materials*.

[44] Choi E., Sul O., Lee J. (2019). Biomimetic tactile sensors with bilayer fingerprint ridges demonstrating texture recognition. *Micromachines (Basel)*.

[45] Gong D., He R., Yu J., Zuo G. (2017). A pneumatic tactile sensor for co-operative robots. *Sensors (Basel)*.

[46] Gastaldo P., Pinna L., Seminara L., Valle M., Zunino R. (2014). Computational intelligence techniques for tactile sensing systems. *Sensors (Basel)*.

[47] Rongala U. B., Mazzoni A., Chiurazzi M. (2019). Tactile decoding of edge orientation with artificial cuneate neurons in dynamic conditions. *Frontiers in Neurorobotics*.

[48] Qin L. H., Yi Z. K., Zhang Y. L. (2018). Unsupervised surface roughness discrimination based on bio-inspired artificial fingertip. *Sensors and Actuators a-Physical*.

[49] Wang Y. C., Chen J. N., Mei D. P. (2020). Recognition of surface texture with wearable tactile sensor array: a pilot study. *Sensors and Actuators a-Physical*.

[50] Zou L., Ge C., Wang Z. J., Cretu E., Li X. (2017). Novel Tactile Sensor Technology and Smart Tactile Sensing Systems: A Review. *Sensors (Basel)*.

[51] Wu C., Kim T. W., Choi H. Y., Strukov D. B., Yang J. J. (2017). Flexible three-dimensional artificial synapse networks with correlated learning and trainable memory capability. *Nature Communications*.

[52] Yang J. J. S., Strukov D. B., Stewart D. R. (2013). Memristive devices for computing. *Nature Nanotechnology*.

[53] Courtland R. (2016). Can HPE's "the machine" deliver?. *IEEE Spectrum*.

[54] Tan H., Tao Q. (2020). Tactile sensory coding and learning with bio-inspired optoelectronic spiking afferent nerves. *Nature communications*.

[55] Wu C., Kim T. W., Park J. H. (2020). Self-powered tactile sensor with learning and memory. *ACS Nano*.

[56] Kim D. W., Yang J. C., Lee S., Park S. (2020). Neuromorphic Processing of Pressure Signal Using Integrated Sensor-Synaptic Device Capable of Selective and Reversible Short- and Long-Term Plasticity Operation. *ACS applied materials & interfaces*.

[57] Kohonen T. (1982). Self-organized formation of topologically correct feature maps. *Biological Cybernetics*.

[58] Mittendorfer P., Cheng G. 3D srface reconstruction for robotic body parts with artificial skins.

[59] Gálvez-García G., De Haan A. M., Lupianez J., Dijkerman H. C. (2012). An attentional approach to study mental representations of different parts of the hand. *Psychological Research-Psychologische Forschung*.

[60] Penfield W., Boldrey E. (1937). Somatic motor and sensory representation in the cerebral cortex of man as studied by electrical stimulation. *Brain*.

[61] Saadon-Grosman N., Ary S., Loewenstein Y. (2020). Hierarchical cortical gradients in somatosensory processing. *Neuroimage*.

[62] Huang Y., Wang L., Li C. Development of a novel fMRI compatible stimulator system for tactile study.

[63] Zeharia N., Hertz U., Flash T., Amedi A. (2015). New whole-body sensory-motor gradients revealed using phase-locked analysis and verified using multivoxel pattern analysis and functional connectivity. *Journal of Neuroscience*.

[64] Jia S., Wang L., Wang H. (2020). Pneumatical-mechanical tactile stimulation device for Somatotopic mapping of body surface during fMRI. *Journal of Magnetic Resonance Imaging*.

[65] Sánchez-Panchuelo R. M., Besle J., Mougin O. (2014). Regional structural differences across functionally parcellated Brodmann areas of human primary somatosensory cortex. *NeuroImage*.

[66] Martuzzi R., van der Zwaag W., Farthouat J., Gruetter R., Blanke O. (2014). Human finger Somatotopy in areas 3b, 1, and 2: a 7T fMRI study using a natural stimulus. *Human Brain Mapping*.

[67] Wang L., Li C., Chen D. (2020). Hemodynamic response varies across tactile stimuli with different temporal structures. *Human Brain Mapping*.

[68] Bensmaia S. J. (2015). Biological and bionic hands: natural neural coding and artificial perception. *Philosophical Transactions of the Royal Society B-Biological Sciences*.

[69] Flesher S. N., Collinger J. L., Foldes S. T. (2016). Intracortical microstimulation of human somatosensory cortex. *Science Translational Medicine*.

[70] Kolasinski J., Makin T. R., Logan J. P. (2016). Perceptually relevant remapping of human somatotopy in 24 hours. *eLife*.

[71] Pugach G., Pitti A., Gaussier P. (2015). Neural learning of the topographic tactile sensory information of an artificial skin through a self-organizing map. *Advanced Robotics*.

[72] McGregor S., Polani D., Dautenhahn K. (2011). Generation of tactile maps for artificial skin. *PLoS One*.

[73] Johansson R. S., Vallbo A. B. (1979). Tactile sensibility in the human hand-relative and absolute densities of 4 types of mechanoreceptive units in glabrous skin. *Journal of Physiology-London*.

[74] Yogeswaran N., Navaraj W. T., Gupta S. (2018). Piezoelectric graphene field effect transistor pressure sensors for tactile sensing. *Applied Physics Letters*.

[75] Bergner F., Dean-Leon E., Cheng G. (2020). Design and realization of an efficient large-area event-driven E-skin. *Sensors*.

[76] de Vignemont F., Majid A., Jola C., Haggard P. (2009). Segmenting the body into parts: evidence from biases in tactile perception. *Quarterly Journal of Experimental Psychology*.

[77] Liu H., Guo D., Sun F. (2016). Object recognition using tactile measurements: kernel sparse coding methods. *IEEE Transactions on Instrumentation and Measurement*.

[78] Disbrow E., Roberts T., Poeppel D., Krubitzer L. (2001). Evidence for interhemispheric processing of inputs from the hands in human S2 and PV. *Journal of Neurophysiology*.

[79] Yau J. M., Kim S. S., Thakur P. H., Bensmaia S. J. (2016). Feeling form: the neural basis of haptic shape perception. *Journal of Neurophysiology*.

[80] Lederman S. J., Klatzky R. L. (2009). Haptic perception: A tutorial. *Attention Perception & Psychophysics*.

[81] da Fonseca V. P., de Oliveira T. E. A., Petriu E. M. (2019). Estimating the orientation of objects from tactile sensing data using machine learning methods and visual frames of reference. *Sensors*.

[82] Moro V., Pernigo S., Tsakiris M. (2016). Motor versus body awareness: voxel-based lesion analysis in anosognosia for hemiplegia and somatoparaphrenia following right hemisphere stroke. *Cortex*.

[83] Huber L., Finn E. S., Handwerker D. A. (2020). Sub-millimeter fMRI reveals multiple topographical digit representations that form action maps in human motor cortex. *NeuroImage*.

[84] Schenk T. (2012). No dissociation between perception and action in patient DF when haptic feedback is withdrawn. *The Journal of Neuroscience*.

[85] Luo S., Mou W., Althoefer K., Liu H. (2019). iCLAP: shape recognition by combining proprioception and touch sensing. *Autonomous Robots*.

[86] Pastor F., Gandarias J. M., García-Cerezo A. J., Gómez-de-Gabriel J. M. (2019). Using 3D convolutional neural networks for tactile object recognition with robotic palpation. *Sensors*.

[87] Mastinu E., Engels L. F., Clemente F. (2020). Neural feedback strategies to improve grasping coordination in neuromusculoskeletal prostheses. *Scientific Reports*.

[88] Okorokova E. V., He Q., Bensmaia S. J. (2018). Biomimetic encoding model for restoring touch in bionic hands through a nerve interface. *Journal of Neural Engineering*.

[89] Osborn L. E., Dragomir A., Betthauser J. L. (2018). Prosthesis with neuromorphic multilayered e-dermis perceives touch and pain. *Science robotics*.

[90] Chandrasekaran S., Nanivadekar A. C., McKernan G. (2020). Sensory restoration by epidural stimulation of the lateral spinal cord in upper-limb amputees. *eLife*.

[91] Loutit A. J., Potas J. R. (2020). Restoring Somatosensation: advantages and current limitations of targeting the brainstem dorsal column nuclei complex. *Frontiers in Neuroscience*.

[92] Ejaz N., Hamada M., Diedrichsen J. (2015). Hand use predicts the structure of representations in sensorimotor cortex. *Nature Neuroscience*.

[93] Shao Y., Hu H., Visell Y. (2020). A wearable tactile sensor Array for large area remote vibration sensing in the hand. *IEEE Sensors Journal*.

